# Xuanhuang Runtong Tablets Relieve Slow Transit Constipation in Mice by Regulating TLR5/IL-17A Signaling Mediated by Gut Microbes

**DOI:** 10.1155/2023/6506244

**Published:** 2023-01-16

**Authors:** Xuejuan Liang, Dan Wan, Yigao Cai, Wei Yue, Xionglong Wang, Huarong Zhou, Rongrong Zhou, Qing Du, Juan Xiao, Shuihan Zhang

**Affiliations:** ^1^Hunan Academy of Chinese Medicine, Changsha, Hunan 410013, China; ^2^Humam Shidai Yangguang Pharmaceutical Co Ltd, Yongzhou, Hunan 425100, China

## Abstract

This study aims to investigate the regulation effects of Xuanhuang Runtong tablets (XHRTs) on intestinal microbes and inflammatory signal toll receptor 5 (TLR5)/interleukin-17A (IL-17A) in STC mice. First, high-performance liquid chromatography (HPLC) was used to verify the composition of XHRT and quality control. Then, the defecation ability of STC mice was evaluated by measuring fecal water content and intestinal transit function. The pathological examination of colonic mucosa was observed by Alcian Blue and periodic acid Schiff (AB-PAS) staining. 16S ribosomal DNA (16S rDNA) genes were sequenced to detect the fecal microbiota. Western blotting, immunofluorescence, and real-time fluorescence quantitative PCR (qRT-PCR) were applied to detect the expression of aquaporin 3 (AQP3), connexin 43 (Cx43), TLR5, and IL-17A. The defecation function of the STC mice was significantly decreased. The amount of mucus secretion and the thickness of the colonic mucus layer were decreased, and the number of microbial species in the intestinal wall, such as Firmicutes/Bacteroidetes, anaerobic bacteria, and *Alistipes*, were also decreased. In addition, the expression of AQP3 and Cx43 was disordered, and the inflammatory factorsTLR5 and IL-17A were activated in the colon. The changes in the above indicators were significantly reversed by XHRT. This study demonstrates that XHRT provides a new strategy for the treatment of slow transit constipation by regulating the activation of the intestinal inflammatory signal TLR5/IL-17A mediated by gut microbes.

## 1. Introduction

Slow transit constipation (STC) is a chronic gastrointestinal disease characterized by intestinal transit dysfunction. The main clinical manifestations of patients include prolonged defecation, reduced defecation frequency, and abdominal distension and pain [1]. The global prevalence of STC is estimated to be as high as 14%, particularly 36% among the elderly. Under severe conditions, STC can cause acute intestinal obstruction or intestinal necrosis perforation and has been identified as a high-risk factor for some gastrointestinal diseases, such as colorectal tumors [2]. The pathogenesis of STC includes inflammation, secretory dysfunctions, gastrointestinal innerves changes, and various abnormalities in gastrointestinal microstructures, the interaction of various factors makes drug treatment difficult [3]. Currently, volumetric, osmotic, or secretory laxatives are commonly used to relieve constipation, but these therapies often fail or have only short-term efficacy and induce side effects [4]. In addition, previous studies indicate that STC surgery has a variable success rate (39–100%), with a high recurrence rate [5]. Therefore, it is very essential to research on a drug that can effectively alleviate and treat STC.

Traditional Chinese medicine (TCM) has a long history of understanding constipation, which was first recorded as “difficulty to defecate” in “Neijing.” STC belongs to the category of “constipation” in TCM, which is one of the dominant diseases treated by TCM. Based on TCM theory, the body's internal heat and a deficiency of body fluid trigger STC. Treatment mainly focuses on invigorating Qi and nourishing Yin, moistening the intestines, and relieving constipation [6]. Various academic publications have reported on the Chinese herbal extracts'effects in treating intractable diseases [7–9], and the effects in treating constipation have also been confirmed [10].

Xuanhuang Runtong tablets (XHRTs) is a prescription composed of 9 Chinese herbal medicines, including *Rehmannia glutinosa* Libosch, *Scrophulariae* radix, *Angelicae sinensis* radix, *Persicae* semen, *Cistanches* herba, *Cannabis* fructus, *Cassiae* semen, and *Aurantii* fructus immaturus. The laxative effect of these Chinese herbal medicines has long been reported in the literature [11–14]. XHRT has a positive curative effect on functional constipation caused by yin deficiency, blood stasis, and senile functional constipation with reasonable safety and few side effects. However, the exact mechanism of action has not been determined.

An increasing number of studies have shown that gastrointestinal dysfunction is closely related to disorders of the host microbiota; a change has been observed in the microbial structure of STC patients, and clinical symptoms have been improved significantly after fecal transplantation [16, 17]. Deng et al. also found that controlling intestinal microbial disorders through a mixture of bacteria could improve loperamide-induced slow transit constipation in rats [18]. Several immunologically active structures protect the mucosal surface of the gastrointestinal tract from antigens and microorganisms [19]. If the intestinal microbiota is disturbed, it can damage intestinal mucosal cells, destroy mechanical and immune barriers, and affect intestinal immunity [20]. One of the key factors of STC may cause abnormal information exchange between the intestinal microbiota and the intestinal mucosal immune system. Intestinal epithelial cells can express a variety of pattern recognition receptors, including toll-like receptors (TLRs) and nucleotide oligomerization domains (NODs). These stimulate intestinal epithelial Th17 cells to produce proinflammatory cytokines, thus directly affecting the intestinal microbiota [21].

Thus, elucidation of the role of intestinal microorganisms in the intestinal immune barrier would provide insights into the mechanism whereby XHRT might be of value in the treatment of STC.

## 2. Materials and Methods

### 2.1. Materials

Xuanhuang Runtong tablets (batch number: 20200101) were purchased from Hunan Shidai Yangguang Pharmaceutical Co., Ltd. (Yongzhou, China). The voucher specimen is stored in the Chinese medicine storage cabinet of the Institute of Chinese Materia Medica, Hunan Academy of Traditional Chinese Medicine. Loperamide hydrochloride capsules (batch number: KFJ2P0P) were purchased from Xi'an Janssen Pharmaceutical Co., Ltd. (Xi'an, China). The reference substances aloin (batch number: 110787-201808, 92.4%), neohesperidin (batch number: 111857-201804, 99.4%), naringin (batch number: 110722-201805, 91.7%), echinacoside (batch number: 111670-201907, 91.8%), and aloe-epine (batch number: 110795-201007, 98%) were purchased from the China Institute for Food and Drug Control.

Alcian Blue and periodic acid Schiff stains were purchased from Visher Corporation (Changsha, China). Rabbit antimouse AQP3 primary antibodies were purchased from ABclonal (Wuhan, China). Cx43 and TLR5 primary antibodies, HRP goat antimouse IgG, HRP goat antirabbit IgG, and coraLite 488-conjugated affiniPure goat antimouse IgG (H + L) were purchased from Proteintech (Chicago, USA). SuperECL Plus was purchased from Advansta (California, USA). The IL-17A primary antibody was purchased from Abcam (Cambridge, UK). TLR5 and IL-17A primers were purchased from Shanghai Shenggong Biological Engineering Co., Ltd. (Shanghai, China).

### 2.2. Animals

Forty male Institute of Cancer Research (ICR) mice (body weight 18∼22 g, age 5∼6 weeks) were purchased from Hunan SJA Laboratory Animal Co., Ltd. (License number: SYXK [Xiang] 2019–0004, animal certificate number: 430727211101222672). Water and food were freely available to the mice under constant temperature, humidity, and light-dark cycles (22 ± 2°C; 55 ± 10%; 07 : 00–19 : 00 light cycle). As mentioned above [22], a mouse STC model induced by loperamide hydrochloride was established with slight modifications. We randomly divided ICR mice into the control group (Control), slow transit constipation (STC) model group, and XHRT high-, medium-, and low-dose (XHRT-H, XHRT-M, and XHRT-L) groups. For 14 days, mice in all groups were gavaged with loperamide hydrochloride solution (10.0 mg/kg) twice a day except for the control group. Different doses of XHRT solution (4.056, 2.028, and 1.014 g/kg, respectively) were administered for 7 days following each administration of loperamide hydrochloride for 1 h in the XHRT-H, XHRT-M, and XHRT-L groups.

### 2.3. Quantitative Analysis of the Main Active Ingredients of XHRT

XHRT was extracted with methanol and filtered through a 0.45 *μ*m microporous membrane. Aloin (0.9235 mg/mL) was dissolved in the mobile phase methanol-0.1% glacial acetic acid aqueous solution (39 : 61). Naringin, neohesperidin, echinacoside, and aloe rhubarb were dissolved in methanol and formulated into reference solutions, with mass concentrations of 0.7561, 0.7510, 0.5196, and 0.5488 mg/mL, respectively.

Quality control of XHRT was conducted using a Dionex UltiMate 3000 series high-performance liquid chromatography instrument (Thermo Fisher Company, USA) with an InertSustain C18 column (5 *μ*m, 4.6 × 250 mm). The column temperature was 25°C, the flow rate was 1.0 mL/min, the injection volume was 10 *μ*L, and the wavelength was 300 nm. The mobile phase contained eluent A (acetonitrile) and eluent B (0.1% formic acid water). The gradient flow was as follows: 0∼10 min: 20%∼40% A; 10∼20 min: 40% A; 20∼30 min: 40%∼42% A; 30∼50 min: 42%∼47% A; 50∼80 min: 47%∼60% A; 80∼90 min: 60%∼90% A; 90∼100 min: 90% A.

### 2.4. Intestinal Transit Function Analysis

On days 0 and 7th of the experiment, the feces of mice were collected and the dry weight of feces was measured to determine whether the model was successfully replicated. The intestinal transit function of the mice was evaluated by a defecation experiment and a small intestine exercise experiment as previously mentioned [22]. On the 14th day, we recorded the time of the first black fecal mass, the number of fecal particles, and the water content within 6 h of the mice (fecal water content = (wet feces weight-dry feces weight)/wet feces weight × 100%). On the 15th day, the small intestine from the pylorus to the ileocecal area of the stomach was collected. Both the length of the small intestine and that of ink advancement were measured to quantify the intestine's transport capacity.

### 2.5. Alcian Blue and Periodic Acid Schiff (AB-PAS) Staining

The proximal colon tissue was fixed in a 4% paraformaldehyde solution, dehydrated by gradient ethanol, and cut into 5 *μ*m thick paraffin sections. It was stained with AB-PAS and sealed with neutral resin. Among them, glycogen- and polysaccharide-positive cells were purple-red and the nucleus was blue. Under an optical microscope (Olympus, Tokyo, Japan), we observed positively stained colonic mucosal epithelial cells and analyzed mucus thickness and secretion.

### 2.6. 16S rDNA Gene Testing

A 16S rDNA gene sequencing technique was used to analyze the community structure of the mouse gut microbiota, as previously described with some modifications [23]. Microbial community genomic DNA was extracted from fecal samples using the E. Z. N. A.® soil DNA kit (Omega Bio-Tek, Norcross, GA, U.S.) according to the manufacturer's instructions. DNA concentration and purity were determined with a NanoDrop 2000 UV-Vis spectrophotometer (Thermo Scientific, Wilmington, USA). The hypervariable region V3-V4 of the bacterial 16S rDNA gene was amplified with primer pairs 338F (5“-ACTCCTACGGGAGGCAGCAG-3”) and 806R (5“-GGACTACHVGGGTWTCTAAT-3”) by TransStart FastPfu DNA Polymerase (ABI, CA, USA). The PCR product was extracted from a 2% agarose gel, purified using the AxyPrep DNA gel extraction kit (Axygen Biosciences, Union City, CA, USA) according to the manufacturer's instructions, and quantified using a Quantus™ fluorometer (Promega, USA).

### 2.7. Microbial Ecology Information Analysis

Purified amplicons were pooled in equimolar amounts and paired-end sequenced on an Illumina NovaSeq PE250 platform (Illumina, San Diego, USA) according to the standard protocols by Majorbio Bio-Pharm Technology Co. Ltd. (Shanghai, China).

After screening the original sequence and removing short sequences (<200 bp), the open reference OTU (operational taxonomic unit) selection method was used to cluster sequences with a similarity >97% into the same operational taxonomic unit (OTU), and then, the classification of species OTUs was performed by homology. The taxonomy of each OTU representative sequence was analyzed by the RDP Classifier (https://rdp.cme.msu.edu/) against the Silva database (SSU123).

The sequencing depth was assessed with the dilution curve generated by OTU clustering. OTU-level alpha diversity indices, such as Chao1, observed species, and Shannon indices, were calculated and analyzed to compare abundance and diversity between samples. A beta diversity analysis was used to examine the structural variation of microbial communities across samples. Based on species with relative abundance >1%, the relative abundance of intestinal mucosal bacteria in mice of different groups was compared at the phylum, family, and genus levels.

### 2.8. Western Blotting Analysis

Protein expressions of AQP3 and Cx43 were detected using western blotting. Total proteins were extracted from colon tissues using RIPA lysis buffer by following the manufacturer's instructions. The protein concentration was determined by using the BCA protein quantification kit. 50 *μ*g of protein was boiled at 100°C for 5 min, separated by SDS-PAGE, and transferred onto PVDF membranes (Millipore). Then, 5% lipid-free milk was used to block the membrane, and the primary antibody diluted with TBST was incubated overnight at 4°C. Primary antibodies targeting AQP3 (A2838; ABclonal), Cx43 (26980-1-AP; Proteintech), and actin (66009-1-Ig; Proteintech) were applied. The membrane was washed with TBST, and the secondary antibody was incubated for 1 h. The hybrid film was developed in a cassette with X-film and exposed for 1–30 min. Then, the protein bands were analyzed by using Quantity One gel analysis software.

### 2.9. Immunofluorescence Analysis

The colon was fixed in 4% paraformaldehyde for 48 h, embedded in paraffin, and cut into 5 mm sections. The sections were baked at 60°C for 12 h and blocked with 10% normal serum/5% BSA, followed by incubation overnight at 4°C in solution with TLR5 (19810-1-AP; Proteintech) and IL-17A (ab79056; Abcam) polyclonal rabbit antimouse antibodies (Proteintech, Wuhan, China), respectively. After washing, the sections were incubated with the goat antirabbit IgG antibody. The sections were then washed again, incubated in the sDAPI working solution at 37°C for 10 min, and were sealed and observed under a laser confocal microscope (ThermoFisher, New York, USA). Three fields (×400) were selected from each section for the positive cell count.

### 2.10. Real-Time Quantitative Polymerase Chain Reaction Analysis

Total RNA in colon tissue was extracted by TRIzol, the OD260/280 value was measured, and the concentration and purity of RNA were calculated. According to TaKaRa's SYBR PrimeScript RT-PCR kit instructions, RNA was reverse-transcribed into cDNA. The reaction system was as follows: 2× SYBR Green PCR Master Mix 10.0 *μ*L, ddH2O 8.5 *μ*L, upstream and downstream primers (10 *μ*mol/L) each 0.5 *μ*L, and cDNA 0.5 *μ*L. The amplification conditions were 95°C for synthase activation for 10 min, 95°C for 15 s, and 60°C for 30 s for 40 cycles. After the reaction, the relative expression levels of TLR5 and IL-17A mRNA were calculated and analyzed by the 2-*δδ*Ct method. Primer sequences were as follows: TLR5 forward 5“-GCCCGTGTTGGTAATATCTC-3,” reverse 5“-ATCTGGGTGAGGTTACAGCCT-3;” IL-17 A forward 5“-TCCACCGCAATGAAGACCCTGA-3,” reverse 5“-TCCAGCTTTCCCTCCGCATTGA-3;” and GAPDH forward 5“-ATCATCTCCGCCCCTTCTG-3,” reverse 5“-GTGATGGCATGGACT GTGG-3”.

### 2.11. Statistical Analysis

Statistical analysis was performed using SPSS 22.0 software (Chicago, IL, USA). The results were expressed as the mean ± standard deviation (SD). One-way ANOVA was used to compare the results (GraphPad Prism 5.0, GraphPad Software, USA); *P* < 0.05 indicated that there were significant differences.

## 3. Results

### 3.1. Quality Control and Identification of XHRT

**Figure 1:**
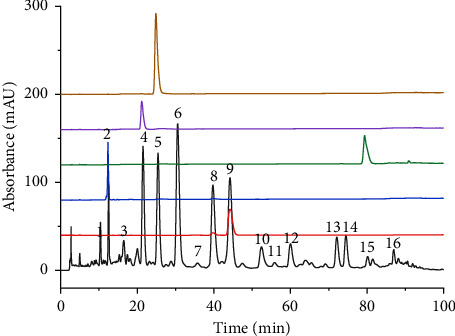
Fingerprints of XHRT and reference substances. (2. Echinacoside, 4. Naringin, 5. Neohesperidin, 9. Aloin, 15. Aloe-Emodin).

The chemical components of XHRT samples were generated by HPLC fingerprints and common peaks by using the similarity evaluation system of traditional Chinese medicine chromatographic fingerprints. Five components, including peak 2 for echinacoside, peak 4 for naringin, peak 5 for neohesperidin, peak 9 for aloin, and peak 15 for aloe-emodin, were analyzed (Figure 1). The contents of echinacoside, naringin, neohesperidin, and aloin were 0.44%, 1.66%, 1.77%, and 1.76%, respectively. The obtained results have shown that our research established a suitable and reproducible chromatographic fingerprint for the quality control of XHRT.

### 3.2. Effect of XHRT on Intestinal Transit Function of STC Mice

**Figure 2:**
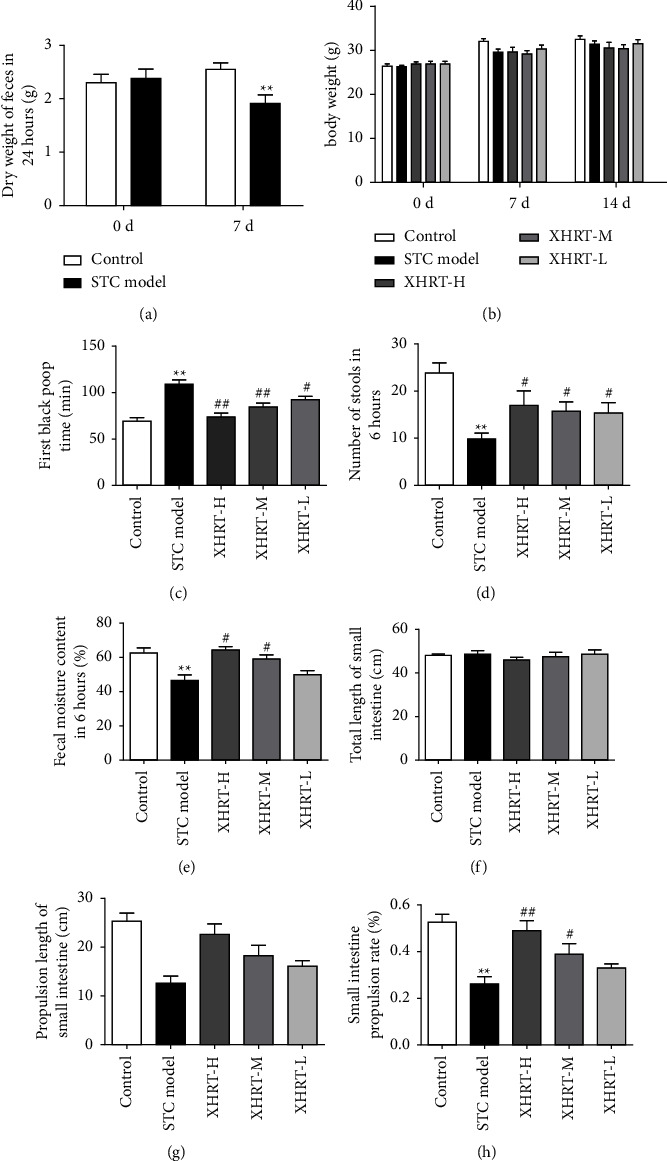
XHRT improved intestinal transit in mice with slow transit constipation induced by loperamide hydrochloride. (a) Comparison of fecal dry weight on day 0 and 7 days after loperamide induction. (b) Changes in body weight of mice at 0 d, 7 d, and 14 d. (c) The effect of XHRT on the time to discharge the first black stool in STC mice, (d) the number of black stools discharged within six hours, and (e) the water content of stool in 6 hours. (f–h) The effect of XHRT on the small intestine transport of STC mice was evaluated by the ink intestinal propulsion exercise. (f) The total length of the small intestine, (g) the length of ink advancing in the small intestine, and (h) the small intestine transport rate. STC: slow transit constipation; XHRT: Xuanhuang Runtong tablet; values are expressed as the mean ± SD, ^*∗∗*^*P*  < 0.01 vs. control group; ^#^*P*  < 0.05, ^##^*P*  < 0.01 vs. STC model group.

We compared the dry weight of feces in the control group and the STC model group at days 0 and 7 and found that the feces of STC mice were dry-knotted and in the form of separated clumps or hard masses of feces. The dry weight of feces at 24 h had a significant reduction (Figure 2(a)), indicating that the model was successfully replicated. After the mice were given different doses of XHRT solution (4.056, 2.028, and 1.014 g/kg), there was no significant difference in body weight (Figure 2(b)). This indicated that XHRT had no effect on the basal metabolism of mice. Compared with the STC model group, the time of the first black stool was significantly reduced and the total amount of black stool and the water content of stool increased within 6h in the XHRT group (Figures 2(c)–2(e)). The small intestine propulsion rate of the STC model group was significantly lower than that of the control group, showing that XHRT significantly improved small intestinal transport (Figures 2(f)–2(h)). These results indicated that XHRT could present a gradient dose-dependent manner to enhance intestinal transit and promote defecation in STC mice.

### 3.3. Effect of XHRT on the Morphological Structure of the Colonic Mucosa in STC Mice

**Figure 3:**
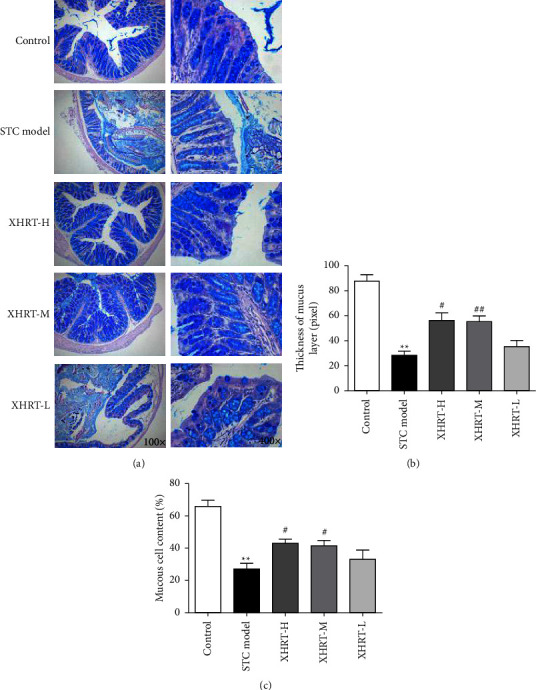
XHRT improved the morphological and structural changes of the colonic mucosa of STC mice treated with loperamide hydrochloride. AB-PAS staining was used to assess the pathogenicity of mouse colon mucosal tissue (a) magnification, 100 times; after XHRT treatment, the thickness of colonic mucosa changed (b) and mucous cell content (c) in STC mice changed; STC: slow transit constipation; XHRT: Xuanhuang Runtong tablet; AB-PAS: Alcian Blue and periodic acid Schiff. Values were expressed as the mean ± SD, ^*∗∗*^*P*  < 0.01 vs. control group; ^#^*P*  < 0.05, ^##^*P*  < 0.01 vs. STC model group.

By staining the colonic epithelial cells with AB-PAS (Figure 3(a)), we found abundant acidic mucins with sulfur mucin and densely distributed goblet cells on both sides of the crypt, with a full and round morphology and a large amount of secreted mucus in the control group. In the STC model group, the mucosal epithelial cells of the colon tissue of the mice showed different degrees of atrophy. There was a reduction in the number of goblet cells, and the acidic mucous layer on the mucosal surface became thinner. In the XHRT-H group, the thickness of the mucous layer on thecolonic mucosa was significantly increased (Figure 3(b)) and the number of goblet cells and mucus secretion significantly increased (Figure 3(c)).

### 3.4. Effect of XHRT on the Intestinal Microbes of STC Mice

#### 3.4.1. Microbial Abundance Change Analysis

**Figure 4:**
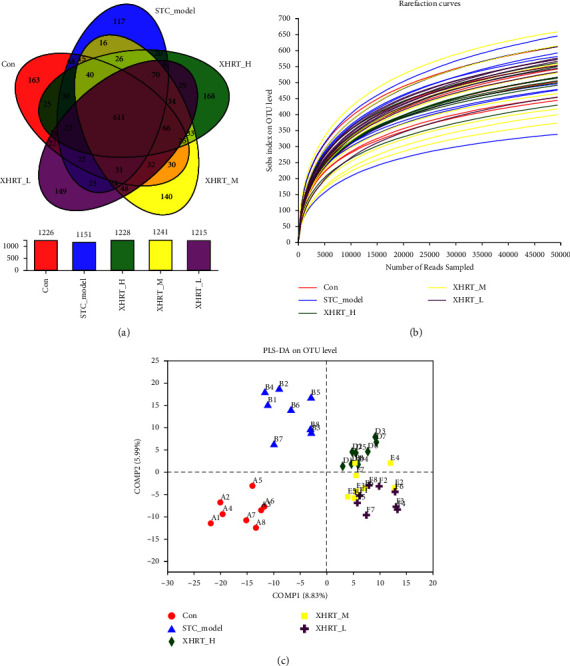
XHRT regulated the structure of the intestinal microbial community in STC mice induced by loperamide hydrochloride. (a) Venn diagram representing the number of OTUs identified in the gut microbiome of STC mice treated with XHRT; mice treated with different doses of XHRT (4.056, 2.028, and 1.014 g/kg) were designated as XHRT-H, XHRT-M, and XHRT-L groups and 16S rDNA microbial gene sequencing. (b) The sparse curve was established based on the Chao1 index of 16S rDNA microbial analysis. (c) PLS-DA analysis based on the full OTUs level. XHRT-H, -M, and -L: Xuanhuang Runtong tablet high dosage, medium dosage, and low dosage. PLS-DA: partial least squares regression analysis method.

To explore the potential participation of the gut microbiota in the effects of the treatment of STC mice with XHRT, we analyzed the community structure of intestinal microbiota in mouse feces using 16S rDNA microbiota profiling. An analysis of the number of operational taxonomic units (OTUs) within the bacterial colony and their crossover between groups was performed using a Venn diagram, and there were 1226 OTUs in the control group, 1151 OTUs in the STC model group, 1228 OTUs in the XHRT-H group, 1241 OTUs in the XHRT-M group, and 1215 OTUs in the XHRT-L group (Figure 4(a)). The sparse curve flattened as the Chao1 index increased, indicating the preferred sequencing depth and high species coverage (Figure 4(b)). There was a significant difference in the abundance of fecal intestinal flora between the STC model group and the control group. After different doses of XHRT treatment, the abundance of intestinal flora in the mice was significantly increased. PLS-DA analysis was performed to estimate the percentage difference in bacterial abundance between samples, and the results showed obvious differences among each group (Figure 4(c)).

#### 3.4.2. Microbial Community Diversity Analysis

**Figure 5:**
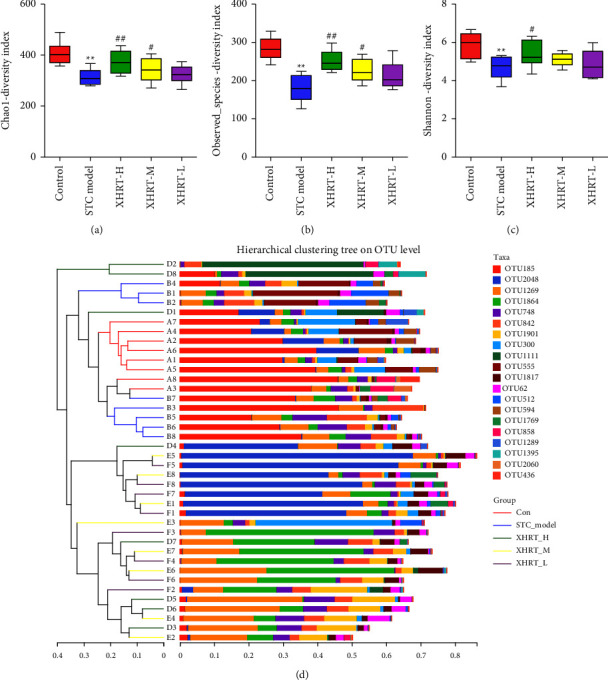
Identification of alpha diversity and beta diversity of the microbiome in STC mice treated with XHRT. (a–c) Microbial abundance in the group was compared based on Chao1 (a), observed species (b), and Shannon (c) indices. (d) The diversity of the microbiome between groups by UPGMA cluster analysis was evaluated. STC: slow transit constipation; XHRT: Xuanhuang Runtong tablet. UPGMA: unweighted pair group method. Values are expressed as the mean ± SD, ^*∗∗*^*P* < 0.01 vs. control group; ^#^*P*  < 0.05, ^##^*P*  < 0.01 vs. STC model group.

The alpha diversity index was used to analyze species diversity in feces, taking into account two factors: the richness and uniformity of species composition. The observed species index of the STC model group was significantly lower than that of the control group. XHRT substantially increased the observed species index, Shannon index, and Chao1 index of the intestinal microbes of STC mice (Figures 5(a)–5(c)), which indicated that XHRT could effectively restore the intestinal microbe alpha diversity of STC mice.

Beta diversity evaluated the differences between microbial communities by comparing the composition of microbial communities. The difference between the samples was analyzed using the unweighted pair group method with arithmetic mean (UPGMA). The similarity between the samples was observed in the distance of branches and the distance of clusters. This resulted in a significant difference between the bacterial communities of the control group and the STC model group (Figure 5(d)). Deviating from the STC model group, the XHRT group was close to the control group. These results indicated that XHRT could improve the composition and diversity of intestinal flora in STC mice.

#### 3.4.3. Microbiota Species Annotation and Difference Analysis

**Figure 6:**
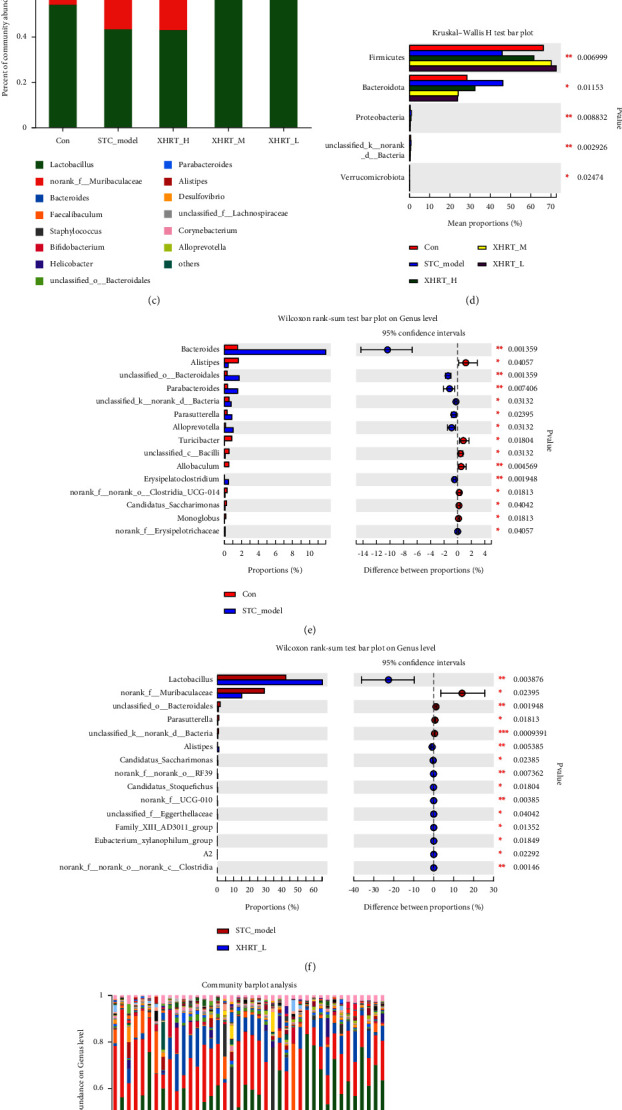
In STC mice treated with XHRT, the relative abundance and family-level composition of the phylum (a), order (b), and genus (c) were assessed. The significance of differences between multiple groups at the phylum level through species differences was analyzed (d). Significant comparison of the difference in bacteria between the two groups and classification levels of intestinal microbes at the genus level (e–g).

At the phylum level, this study identified Firmicutes, Bacteroides, Actinomycetes, *Campylobacter*, and Desulfobacterota from 40 samples. The relative abundance of Firmicutes and Bacteroides was relatively high, and the ratio of Firmicutes/Bacteroidetes (F/B) reflected the health status of the body. In comparison with the control group, its relative abundance significantly decreased in the STC model group but recovered with XHRT treatment (Figures 6(a) and 6(d)). At the order level, *Lactobacillus* abundance in the STC model group was significantly lower than that in the control group. In contrast, *Lactobacillus* abundance in the XHRT group was higher than that in the STC group (Figure 6(b)). At the genus level, we found that the relative abundance of *Lactobacillus* and norank_f_*Muribaculaceae* in the STC model group decreased and increased after XHRT intervention compared with the control group (Figures 6(c), 6(e), and 6(f)). We obtained a total of 135 bacterial genera from the samples, mainly *Lactobacillus*, norank_f_*Muribaculaceae*, *Bacteroides*, *Faecalibaculum*, and *Staphylococcus* (Figure 6(g)), covering more than 70% of all microbial species. These results suggested that the changes in the intestinal microbial community structure during the pathogenesis of STC may be reversed by XHRT treatment.

### 3.5. Effect of XHRT on the Expression of Protein AQP3 and Cx43 in STC Mice

**Figure 7:**
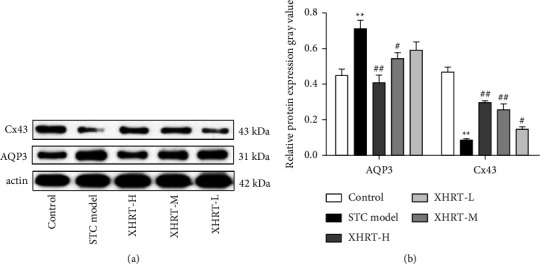
XHRT regulated the expression of colonic aquaporin AQP3 and basal-related protein Cx43 in the colonic mucosa of STC mice induced by loperamide hydrochloride. Western blotting was used to detect and analyze the expression of AQP3 and Cx43 in mouse colon tissue (a). Quantity One software analyzed protein gray bands and counted relative protein expression (b). STC: slow transit constipation; XHRT: Xuanhuang Runtong tablet; AQP3: aquaporin 3; Cx43, gap junction protein. Values were expressed as the mean ± SD, ^*∗*^*P*  < 0.05, ^*∗∗*^*P*  < 0.01 vs. control group; ^#^*P*  < 0.05, ^##^*P*  < 0.01 vs. STC model group.

Western blot analysis showed that the relative gray value of Cx43 protein was significantly reduced in the colon tissue of the STC model group, while that of AQP3 protein was significantly increased compared with the control group. After XHRT treatment, the relative gray values of AQP3 and Cx43 protein were reversed (Figures 7(a) and 7(b)). As a result of XHRT, colon feces in STC contained more water and promoted defecation, which was closely related to the expression of water secretion protein AQP3 and grassroot-related protein Cx43.

### 3.6. Effect of XHRT on the Expression of Intestinal Mucosal Immune Barrier Regulation-Related Proteins and Genes in STC Mice

**Figure 8:**
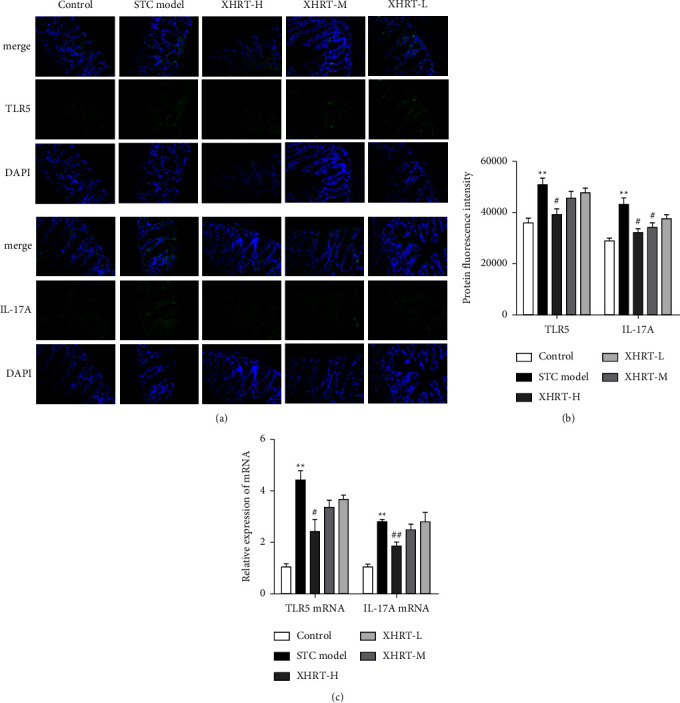
XHRT regulated the expression of TLR5/IL-17A related to the intestinal mucosal immune function in the STC mice induced by loperamide hydrochloride (a).Immunofluorescence method used antibodies targeting TLR5 and IL-17A proteins, observed the protein localization and fluorescence intensity under a laser confocal microscope, and used the average fluorescence intensity of the protein to reflect the relative expression level of each protein (b) (400×). (c) The relative expression of TLR5 mRNA and IL-17A mRNA in colon tissue by real-time fluorescent quantitative PCR with GAPDH as an internal reference was detected. STC: slow transit constipation; XHRT: Xuanhuang Runtong tablet; TLR5: toll receptor 5; IL-17A: interleukin-17A; GAPDH: glyceraldehyde 3-phosphate dehydrogenase. Values were expressed as the mean ± SD, ^*∗∗*^*P*  < 0.01 vs. control group; ^#^*P*  < 0.05, ^##^*P*  < 0.01 vs. STC model group.

Using immunofluorescence (IF), we determined the expression of mouse colon tissue protein TLR5 and Th17 cell-related factor IL-17A to further investigate the molecular mechanism of XHRT on defecation and intestinal mucosal immune function in STC mice. Using laser confocal microscopy, we observed the localization of each protein and the fluorescence intensity (Figure 8(a)). Compared with the control group, the fluorescence intensity of TLR5 and IL-17A proteins in the colon tissue of the STC model group was increased. The relative fluorescence intensity of TLR5 and IL-17A proteins in posterior colon tissue was significantly reduced by XHRT administration (Figure 8(b)).

At the same time, we detected the expression of TLR5 and IL-17A mRNA in colon tissue by qRT-PCR (Figure 8(c)) and found that the relative expression of TLR5 and IL-17AmRNA in the STC model group significantly increased compared with that in the control group. Based on the abovementioned protein detection trend, the relative expression of TLR5 and IL-17A mRNA in the colon tissues of mice in the XHRT-H was significantly reduced. These results revealed that XHRT could effectively modulate the immune function of the intestinal mucosa by regulating the expression of the intestinal TLR5/IL-17A signaling pathway.

## 4. Discussion

Slow transit constipation is one of the most common types of refractory functional constipation being studied by scholars. The pathogenesis of STC involves multiple factors, but little is known about its pathophysiology and etiology. Among them, colonic sensory motor dysfunction and decreased sensitivity are the most widely recognized causes. As a result of these abnormal changes, the intestinal mucosal barrier is unable to maintain its normal structure and function for a prolonged period [24]. Intestinal mucosal barriers include mechanical, biological, and immune barriers, which interact to play a synergistic role. In current studies, it has been found that patients with functional constipation experience changes in intestinal mucosal permeability. These changes participate in the pathogenesis of constipation by influencing intestinal motility, intestinal sensitivity, and intestinal immune status [25]. However, the underlying molecular mechanism remains unclear.

As the first barrier of intestinal mucosal defense, the mechanical barrier consists of intestinal epithelial cells, mucins, electrolytes, and water on the surface of the intestinal lumen, lubricating the intestine and providing a habitat for intestinal commensal bacteria. In patients with constipation, there is a significant decrease in the secretion of goblet cells (mainly secreting mucins) in the colonic mucosa and the thickness of the mucus layer is reduced, which was also confirmed in our study.

The structural basis of the mechanical barrier is mainly composed of junctional complexes between intestinal epithelial cells. The role of claudin in maintaining the structure of the intestinal mucosal mechanical barrier in patients with constipation has been reported in several studies [26, 27]. A class of connexins found in colonic epithelial cells is closely related to gastrointestinal slow waves and gastrointestinal motility disorders. Among them, abnormal expression of Cx43 may cause gastrointestinal diseases, such as gastrointestinal tumors, Hirschsprung's disease, and functional dyspepsia [28, 29]. A study by Bhave et al. [30] revealed that the expression of Cx43 protein in the intestinal mucosa of functionally constipated patients was significantly reduced. This occurred because its abnormal expression could cause changes in the intestinal mucosa and inflammation. The regulation of intestinal water fluid is an important manifestation of mechanical barrier function. Epithelial cells of the colonic mucosal villi have aquaporin AQPs located at the top and bottom of their plasma membranes, which are involved in the transmembrane transport of water. This plays a pivotal role in the regulation of intestinal absorption, secretion, and water metabolism [31]. When the expression level of its critical isoform AQP3 increases, the intestinal contents absorb too much water, causing the accumulation of colonic contents, which can lead to difficulty in defecation [32]. Our study shows that XHRT could significantly improve the structural and molecular changes in the colonic mucosal mechanical barrier in STC mice. Further research was conducted to determine whether these were related to the synergistic regulation of the biological barrier and immune barrier.

According to a literature survey, the gut bacteria *B. thetaiotaomicron* and *Faecalibacterium prausnitzii* promote goblet cell differentiation and induce mucin glycosylation-related genes in the biological barrier [33]. Using the active substance of sennoside A metabolized by the intestinal flora, Kon et al. [34] demonstrated that macrophages secrete PGE2. This reduces AQP3 expression in the colonic epithelium of rats, prevents the colon from absorbing water, and promotes defecation. Based on the available evidence, we can speculate that XHRT modulates the structure and function of the mechanical barrier while altering the composition and metabolism of gut microbes symbiotic in the mucus layer, thereby affecting defecation.

Gut microbes constitute a biological barrier against pathogens. In recent years, breakthroughs in the field of intestinal microbiology have provided new strategies for the diagnosis and treatment of slow transit constipation. Due to changes in the modern lifestyle and diet structure, the composition and diversity of the intestinal microbiota have changed, causing symptoms such as constipation. Additionally, constipation can aggravate the imbalance of intestinal microbes and even cause colon cancer, accelerate aging, and promote a variety of intestinal diseases [2, 3, 35]. Studies have found that compared with healthy controls, the number and quality of beneficial bacteria (such as *Lactobacillus*, *Bifidobacterium*, and *Bacteroides*) in the colonic mucosal microbiota of STC patients are significantly reduced. The number of potential pathogens (such as verdigris *Pseudomonas*, *Campylobacter jejuni*, and *Clostridium putrefaction*) increased significantly [25, 36]. Of note, these changes in the composition of intestinal microbes may affect intestinal motility and alter local environmental homeostasis [37].

Our study analyzed the composition, structure, and diversity of intestinal microbial communities using 16S rDNA high-throughput sequencing in STC model mice. The ratio of Firmicutes/Bacteroidetes closely associated with constipation was significantly reduced. Moreover, *Alistipes*, a bacterial anaerobic bacterium closely associated with intestinal inflammation, was found to be less abundant. In addition to increasing the abundance of intestinal microorganisms, XHRT also increased the abundance of lactic acid bacteria and *Alistipes*. Several studies reported that patients taking probiotics for anti-inflammatory purposes had higher *Alistipes* levels [38]. The impact of *Alistipes* on intestinal immunity was researched by Shi et al. [39] in 2020, but it was rarely reported in STC patients. To date, no consensus has been reached regarding the characteristics of the gut microbiota of STC patients.

The results of our study suggest that unregulated migration of gut microbes into the lamina propria may be caused by disruption of the intestinal mucosal mechanical barrier and increased permeability in patients with STC. Pathogenic microorganisms (such as pathogenic *Escherichia coli*) release surface antigens that are recognized by intestinal antigen receptors, which amplify inflammation.

Currently, there are limited studies on the intestinal mucosal immune barrier in STC patients. We found that intestinal immune cells can regulate intestinal injury and inflammation by recognizing indigestible dietary components such as fiber decomposed by microorganisms and producing metabolites [40]. The cell wall components of Gram-positive bacteria (*Bifidobacterium* or *Lactobacillus*) can induce an increase of important phagocytosis receptors(such as Fc*γ*RIII and TLR), and then, primary T cells respond to cytokines produced in adaptive immunity to distinguish specific CD4 + T helper subtype cells (Th1, Th2, or Th17) [41]. Some gut microbes play protective roles against immune responses, promoting tissue repair and survival in part through toll-like receptor (TLR) activation [42]. The goblet cells in the mechanical barrier mucous layer prevent direct contact between gut microbes and pattern recognition receptors in epithelial cells, which may trigger an immune response. XHRT can significantly increase the thickness of the acidic mucus layer on the surface of the colonic mucosa, which provides a stable breeding place for commensal bacteria, thus reducing the occurrence of inflammatory reactions.

As a member of the TLR family, TLR5 is closely related to the immune response in the intestinal mucosa. It has high expression in the lamina propria of CD11c-positive intestinal cells and activates inflammation in response to conditional pathogens such as *Escherichia coli* and *Enterococcus* [43]. In addition, fecal microbial transplantation (FMT) stimulates the intestinal adaptive immune response through the TLR pathway, thereby promoting the synthesis of immunoglobulins (such as IgA, IgG, and IgM) and protecting the intestinal mucosa [44]. *Alistipes* in the gut is closely associated with the production of inflammatory factors induced by TLR receptors [45]. At the same time, the intestinal mucosa is the natural site for the differentiation and maturation of helper T cells 17. Th17 cells can secrete interleukin 17, and TLR5 signaling can activate the high expression of IL-17A in the mucosa, thereby triggering intestinal immunity [17]. *Bifidobacterium adolescentis* and segmental filamentous bacteria have been identified as effective inducers of IL-17-producing Th17 cells [46, 47]. The expansion of Th17 cells is usually related to the destruction of intestinal microbiome homeostasis and the impairment of *T* regulatory cells. A strong induction of the intestinal microbiota of Th17 cells can aggravate colonic inflammation in mice [48].

Based on the interaction between intestinal microbes and the TLR5/IL-17A signaling pathways in intestinal mucosal immunity, we located and quantitatively analyzed TLR5/IL-17A protein in the colon tissue of STC mice using immunofluorescence. We found that XHRT significantly regulated the TLR5/IL-17A signaling pathway in colon mucosal inflammation in STC mice. Additionally, the qRT-PCR method was used to perform double verification at the genetic level, revealing the molecular mechanism behind intestinal microbial diversity change and intestinal mucosal immunity disorders in STC. The results of our research demonstrate how XHRT contributes to the nourishment of the intestine and defecation. They also provide new experimental insights into clinical medicine by differentially regulating intestinal microbes, intestinal mucosal immunity, and signal transduction pathways. Throughout the research, multipathway and multitarget regulation have been observed in Chinese herbal medicine. Our studies will continue to clarify the direct relationship between the host metabolism of STC intestinal commensal bacteria and intestinal mucosal immune function.

## 5. Conclusions

This study has demonstrated that regulating the activation of intestinal inflammatory signal TLR5/IL-17A mediated by gut microbes can improve slow transit constipation and that XHRT provides a new strategy for the treatment of slow transit constipation.

## Data Availability

The data used to support the findings of this study are included within the article.

## Abbreviations

STC: Slow transit constipation
XHRT: Xuanhuang Runtong tablet
TLR5: Toll receptor 5
IL-17A: Interleukin-17A
16S rDNA: 16S ribosomal RNA
AQP3: Aquaporin3
Cx43: Connexin 43
qRT‒PCR: Real-time fluorescence quantitative PCR
TCM: Traditional Chinese medicine
OTUs: Operational taxonomic units
ICR: Institute of Cancer Research
ECL: Chemiluminescence.
